# Supplementary material for: Effects of Piptoporus betulinus Ethanolic Extract on the Proliferation and Viability of Melanoma Cells and Models of Their Cell Membranes
Source: Int J Mol Sci. 2022 Nov 11;23(22):13907. doi: 10.3390/ijms232213907 (PMC9692381; doi:10.3390/ijms232213907)
Supplement: Supplementary file 1 [file ijms-23-13907-s001.zip › ijms-1995665-supplementary.pdf]

Supporting information

Effects of *Piptoporus betulinus* ethanolic extract on the proliferation and viability of melanoma cells and models of their cell membranes

Justyna Bożek[1]*, Joanna Tomala[1], Sylwia Wójcik[1], Beata Kamińska[1], Izabella Brand[2], Ewa Pocheć[3], Elżbieta Szostak[1]

[1]*Faculty of Chemistry, Jagiellonian University, Gronostajowa 2, 30-387 Krakow, Poland*

[2]*Department of Chemistry, Carl von Ossietzky University Oldenburg, Oldenburg, Germany*

[3] *Department of Glycoconjugate Biochemistry, Institute of Zoology and Biomedical Research, Faculty of Biology, Jagiellonian University, Kraków, Poland*

Contents

S1. Determination of TPC S2

S2. Determination of AEAC S3

S3. Composition and packing of lipids in the three-component model membrane S4

S4. Langmuir isotherms of the DMPC bilayer S5

S5. IR absorption modes of the *Piptoporus betulinus* extract S6

S6. PM IRRA spectra of the DMPC bilayers S7

S1. Determination of TPC

Table S1 presents a set of data obtained during measuring UV/VIS absorption spectra of gallic acid standard solutions.

Table S1. The mean value and standard deviation of absorbance measured at 765 nm for gallic acid solutions.

Concentration [mg/ml]	Absorbance
0.05	0.030 ± 0.001
0.1	0.095 ± 0.001
0.15	0.141 ± 0.001
0.2	0.184 ± 0.001
0.25	0.245 ± 0.001
0.3	0.271 ± 0.001
0.35	0.356 ± 0.002
0.4	0.373 ± 0.001
0.45	0.433 ± 0.001
0.5	0.481 ± 0.001

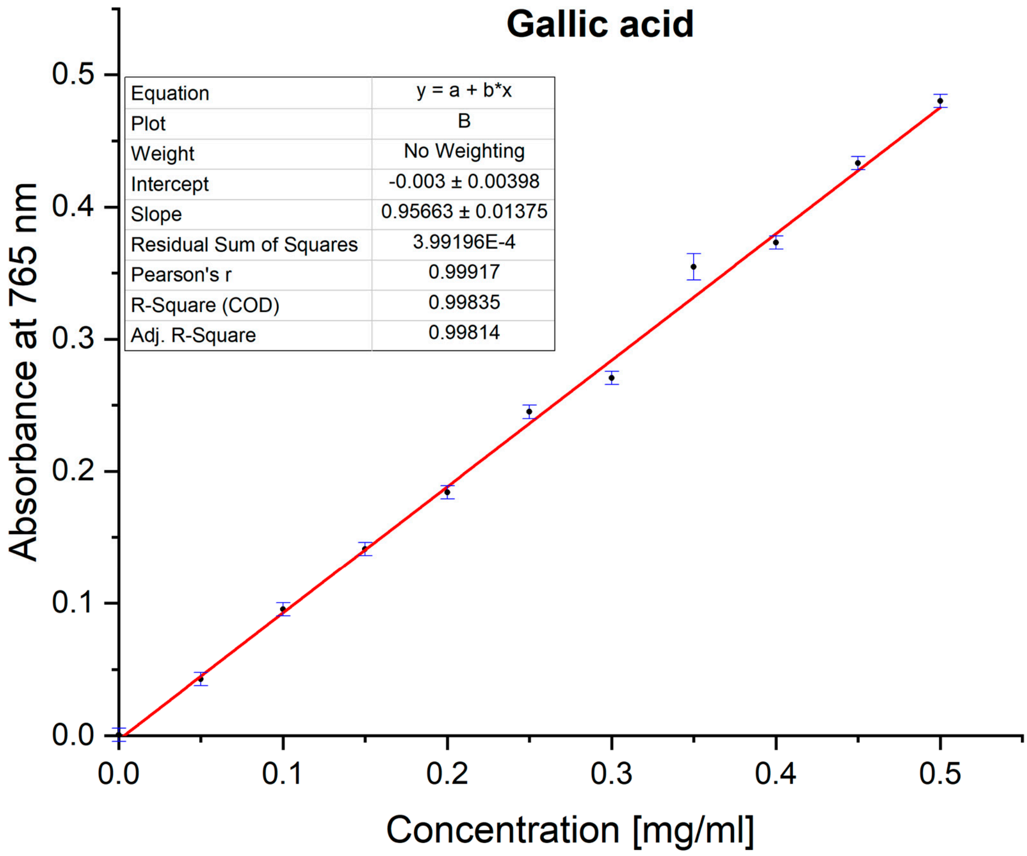

Figure S1. Gallic acid standard calibration curve.

S2. Determination of AEAC

Table S2 presents a set of data obtained during measuring UV/VIS absorption spectra of ascorbic acid standard solutions.

Table S2. The mean value and standard deviation of absorbance measured at 750 nm for ascorbic acid solutions.

Concentration [µg/ml]	Absorbance
10	0.373 ± 0.002
20	0.759 ± 0.002
40	1.493 ± 0.002
60	2.100 ± 0.001
80	2.694 ± 0.002
100	3.434 ± 0.002

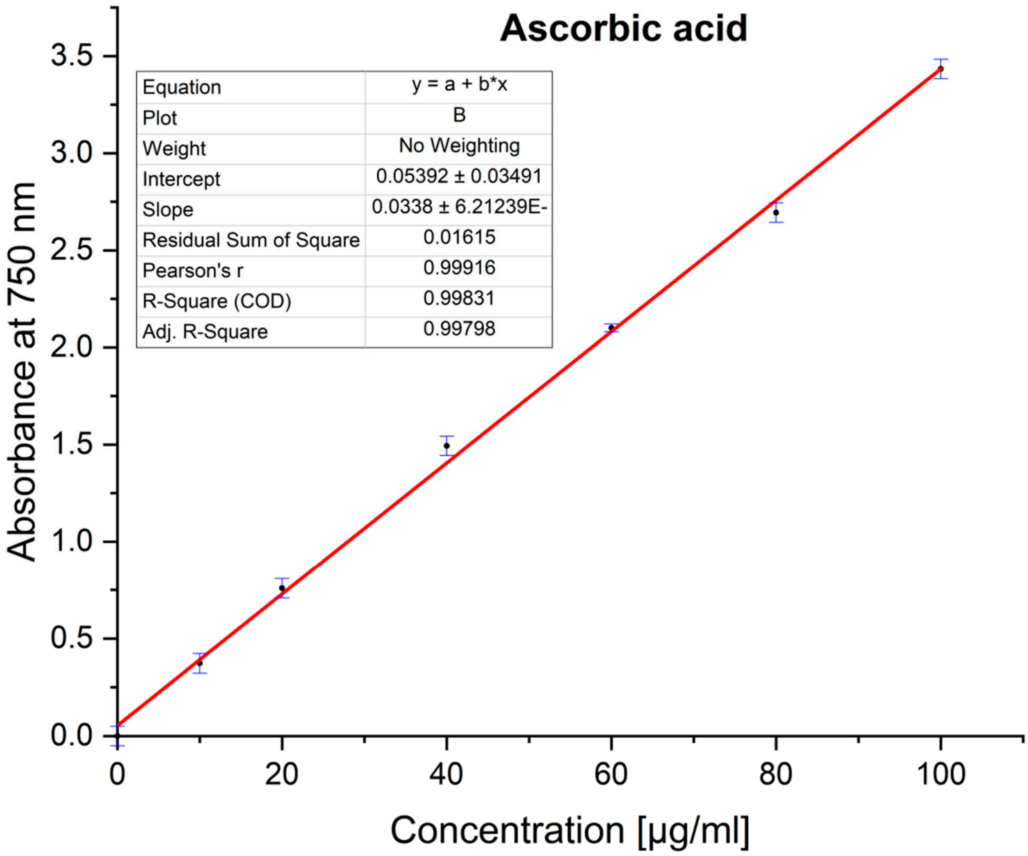

Figure S2. The calibration curve determined for ascorbic acid.

S3. Composition and packing of lipids in the three-component model membrane

The inner, Au surface-facing leaflet of the multicomponent asymmetric lipid bilayer contained DMPC and cholesterol (7:3 mole ratio) and the outer leaflet contained DMPC, GD_{1a} gangliosides and cholesterol (5:2:3 mole ratio). A schematic picture of the bilayer structure is shown in Figure S3.

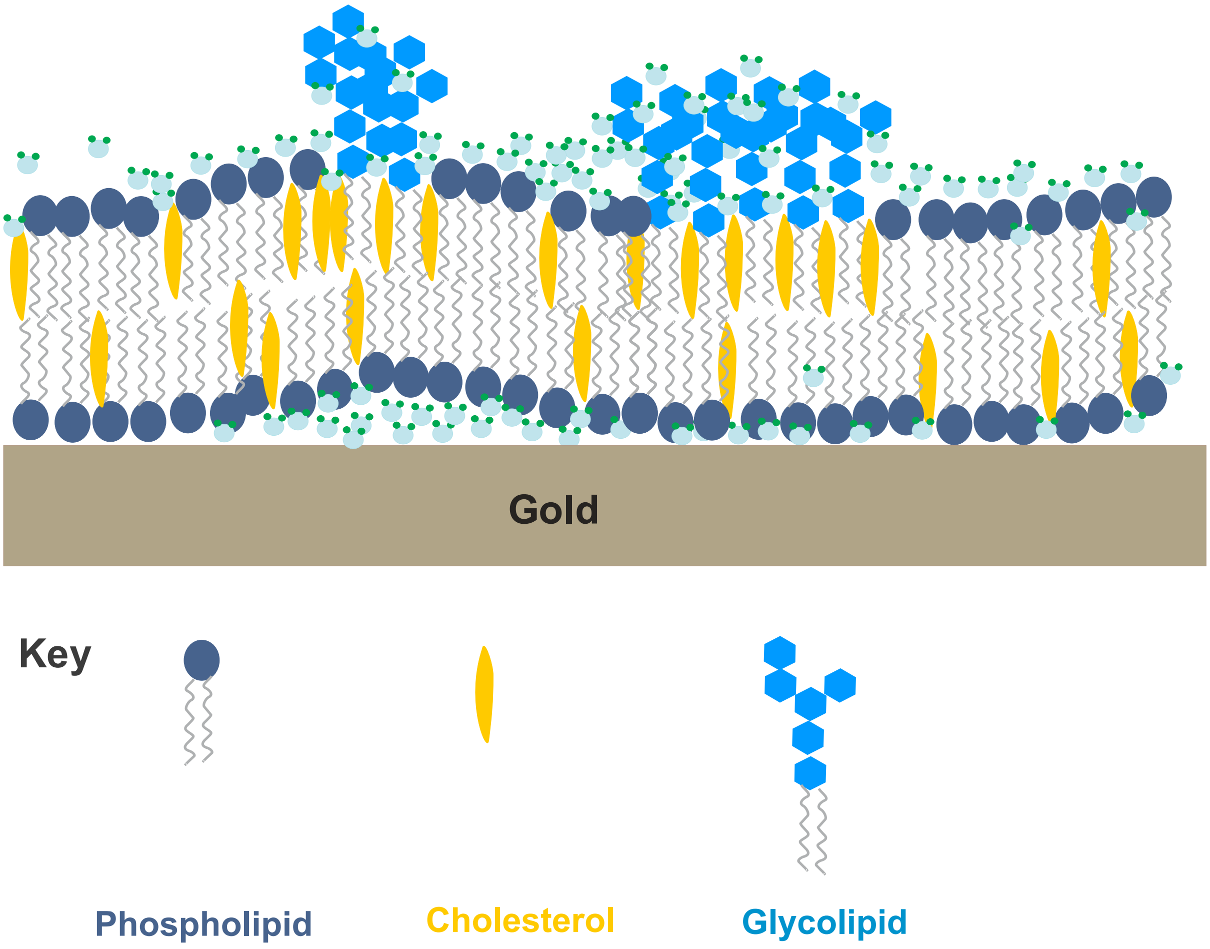

Figure S3. Composition and packing of lipids in the DMPC:chol–DMPC:GD_{1a}:chol model membrane.

S4. Langmuir isotherms of the DMPC bilayer

Langmuir isotherms of the DMPC bilayer and after interaction with the *Piptoporus betulinus* extract are shown in Figure S4. Inset shows the compressibility modulus plots as a function of the surface pressure.

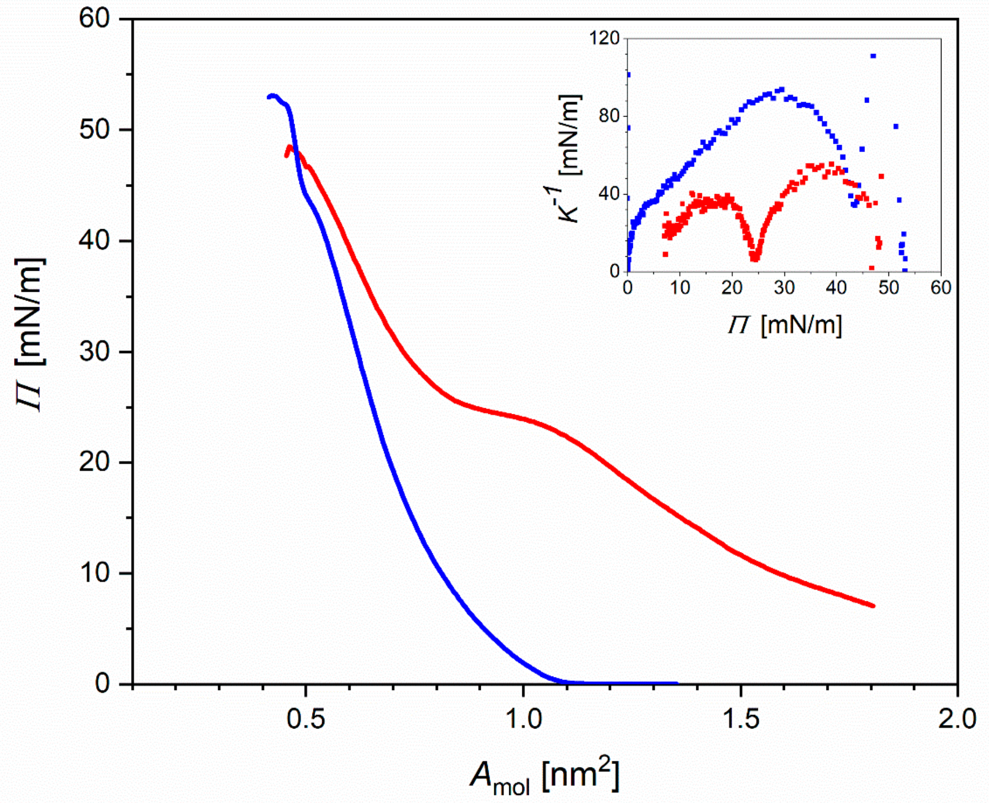

Figure S4. Surface pressure *Π* versus area per molecule (A_{mol}) isotherms of the DMPC monolayer on the aqueous (blue) and 1% aq. *Piptoporus betulinus* extract after 30 minutes of incubation (red). Inset: Compressibility modulus versus *Π* of the lipid monolayer on the aqueous (blue) and 1% vol *Piptoporus betulinus* ethanolic extract in water after 30 minutes of incubation (red).

S5. IR absorption modes of the *Piptoporus betulinus* extract

Table S3 lists the wavenumbers of the deconvoluted IR absorption modes in the 1820 – 1500 cm^{-1} spectral region.

Table S3. Assignment of the IR absorption modes in the IR spectrum in the 1820 – 1500 cm^{-1} spectral region of the dried *Piptoporus betulinus* extract.

Wavenumber / cm^{-1}	Assignment
1743	ν(C=O) aldehydes, esters
1735	ν(C=O) aldehydes, esters
1713	ν(C=O) ketones, carboxylic acids
1703	ν(C=O) ketones, carboxylic acids
1672	ν(C=O) conjugated, cyclic and aromatic ketones, esters; $\nu_{as}(COO^-)$
1642	Amide I; ν(C=C)aliphatic (R5 ring in botulin)
1612	$\nu(CC)_{i.p.}$aromatic; ν(C=N)aliphatic
1583	$\nu(CC)_{i.p.}$aromatic; ν(C=N)aromatic, amide II
1555	$\nu(CC)_{i.p.}$aromatic; amide II

S6. PM IRRA spectra of the DMPC bilayers

Figure S5a shows the ATR spectrum of the *Piptoporus betulinus* extract in the 1820 – 1500 cm^{-1} spectral region. Figures S5b and c show the deconvoluted PM IRRA spectra of the DMPC bilayer after interaction with the *Piptoporus betulinus* extract (S5b) and lipid membrane (S5c).

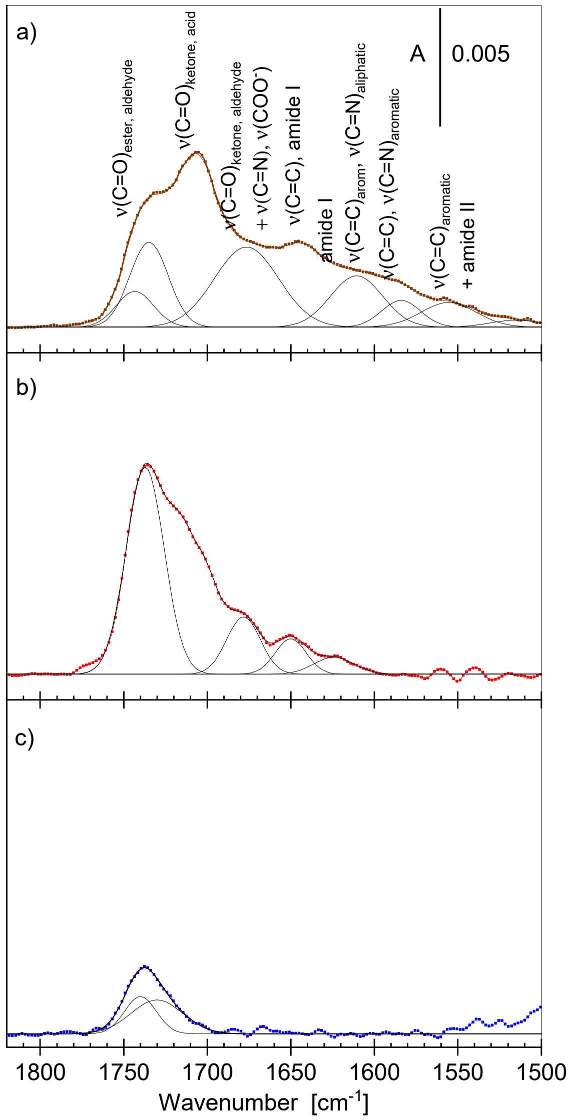

Figure S5. a) Deconvoluted ATR IR spectrum (brawn line) of a dried extract from *Piptoporus betulinus* and b,c) deconvoluted PM IRRA spectra (red and blue lines) of the DMPC bilayer on the Au surface transferred from b) 1% vol *Piptoporus betulinus* extract

aqueous subphase (red) and c) aqueous (blue) subphase in the 1820 – 1500 cm^{-1} spectral region.
